# The Oral Microbiome in Queensland Free-Ranging Koalas (*Phascolarctos cinereus*) and Its Association with Age and Periodontal Disease

**DOI:** 10.3390/ani15131834

**Published:** 2025-06-20

**Authors:** Lyndall Pettett, Esmaeil Ebrahimie, Teerapol Chinkangsadarn, Manijeh Mohammadi Dehcheshmeh, Darren J. Trott, Philip S. Bird

**Affiliations:** 1 School of Veterinary Sciences, Faulty of Science, The University of Queensland, Gatton, QLD 4343, Australia; phil.bird@uq.edu.au; 2 Genomics Research Platform, School of Agriculture, Biomedicine and Environment, La Trobe University, Melbourne, VIC 3086, Australia; 3 Australian Centre for Antimicrobial Resistance Ecology, School of Animal and Veterinary Sciences, The University of Adelaide, Roseworthy, SA 5116, Australia; manijeh.mohammadidehcheshmeh@adelaide.edu.au (M.M.D.); darren.trott@adelaide.edu.au (D.J.T.); 4 Faculty of Veterinary Science, Chulalongkorn University, Bangkok 10330, Thailand; teerapol.chin@gmail.com

**Keywords:** koala, microbiome, oral health, gingivitis, periodontitis, age, periodontal disease

## Abstract

This study was developed to profile the oral microbiome of free-ranging Queensland koalas and its association with age, gingivitis and periodontitis. Using next-generation sequencing of 16S rRNA genes, the microbiota of oral plaque samples from eight koalas across different age groups (joey, juvenile, adult and old) were compared. The findings revealed significant shifts in microbiota composition with age and disease presence. At the phylum level, Proteobacteria were the most dominant phylum, especially in younger koalas. Proteobacteria abundance decreased with age, while Bacteroidetes, Fusobacteria and Actinobacteria increased. At the genus level, *Acinetobacter* declined with age. *Fusobacterium* and *Porphyromonas* became more prominent genera in older koalas and those with periodontal disease. The beneficial genus *Lactobacillus* was detected only in the joey, suggesting a potential loss of protective microbes with age. Alpha diversity analysis showed high variability within individuals based on age. Alpha diversity was remarkably lower in younger koalas and increased with periodontal disease. Beta diversity suggested distinct microbiota composition differences between younger (joey and juvenile) and older (adult and old) koalas, although statistical significance was limited by sample size. This is the first detailed characterization of the oral microbiome in Queensland’s free-ranging koalas and highlights its association with age and oral health status. Findings may contribute to better understanding of oral disease progression in koalas and support conservation and health management efforts.

## Simple Summary

The koala (*Phascolarctos cinereus*), a marsupial native to Australia, is facing serious population decline, partly due to systemic and oral health issues. This study provides the first detailed characterization of the oral microbiome in free-ranging koalas from Queensland using 16S rRNA gene sequencing of oral plaque from individuals across four age groups. Significant age-related shifts in microbial composition were observed. At the phylum level, younger koalas had higher levels of Proteobacteria, while Bacteroidetes, Fusobacteria and Actinobacteria increased with age. At the genus level, older koalas and those with periodontal disease exhibited significantly higher abundances of *Fusobacterium* and *Porphyromonas*. Notably, the beneficial genus *Lactobacillus* was detected only in the joey, suggesting a potential loss of protective microbes with age. These findings reveal distinct microbial patterns associated with age and oral disease, offering valuable insights into koala health and conservation.

## 1. Introduction

The koala (*Phascolarctos cinereus*) is a marsupial endemic to Australia. The koala is listed as vulnerable under the EPBC Act with populations declining rapidly in New South Wales and Queensland. Primary threats to the koala are vegetation reduction, motor vehicles, dog attacks and disease. The current major disease threats are chlamydia caused by *Chlamydia pecorum* and the koala retrovirus [1]. The retrovirus has been identified as having links to other diseases such as neoplasms [2]. Studies on oral diseases of koalas have identified periodontal disease as another threat on koala health [3,4,5,6,7]. In a recent study of koalas with ocular discharge from a *C. pecorum* infection, it was considered as an indication of an early clinical sign of dental disease, and it was suggested that CT would enable detection of lacrimal canal abnormalities secondary to dental disease [8]. Periodontal diseases, gingivitis and periodontitis (PD) are chronic inflammatory diseases initiated by bacteria in the plaque [9]. Gingivitis is a reversible condition which involves the polymicrobial biofilm in the gingival sulcus, resulting in bleeding, redness and swelling of the gingivae. Periodontitis is an irreversible condition, resulting in loss of gingival attachment with the formation of pockets, reabsorption of the supporting alveolar bone with increasing tooth mobility and eventual tooth loss. Evidence indicates that periodontal disease in koalas follows the same pathway of periodontal destruction as in other species [3] with *Porphyromonas* strains from the captive marsupial oral cavity suggested to have a role in PD [10]. Koalas do have one unique contributory characteristic in the progression on alveolar bone loss whose role in the pathogenic pathway has not been fully explained. During the mastication of the tough eucalyptus leaves, pulp material compacts into interdental spaces, becoming odorous as it undergoes the rotting process, making ideal conditions for further gingivae and bone destruction [11].

The oral cavity is regarded as having the second largest diverse microbiota ecosystem in the body [12]. Associations between the oral and gut microbiota have been observed, with suggestions that the microbiota of the oral cavity influences the gut microflora taxonomy [12]. Additionally, there are links between oral inflammatory conditions such as PD and systemic health such as diabetes mellitus, pneumonia and heart disease [13]. Good oral health is predominantly characterized by the presence of Gram-positive bacteria, fungi, viruses and protozoa [14], while in PD there is a shift to a more anaerobic Gram-negative microbiota [15].

Oral microbiomes in mammals and marsupials commonly have varying ratios of Proteobacteria, Bacteroidetes, Firmicutes, Actinobacteria and Fusobacteria, whether they are herbivores or carnivores [16,17,18]. In a study of the tammar wallaby (*Macropus eugenii*) saliva, 48 unique phylotypes were identified, and the phylum Proteobacteria represented 54.2% of the phylotype diversity [17]. In a recent study, the microbiota of macropods detected 28 phyla with Proteobacteria and Actinobacteria as the most abundant, with healthy and periodontitis-osteomyelitis samples distinguished from one another through microbial shifts where *Porphyromonas*, *Fusobacterium* and *Bacteroides* were the primary antagonists in periodontal disease [19]. The oral microbiome of two captive koalas revealed that Proteobacteria and Bacteroidetes represented 90% of the bacterium discovered, while the remaining 10% belonged to the Firmicutes phylum [18]. Furthermore, microbial research into the oral cavity of the koala has led to the identification of a novel microbial species [20,21].

Many microbes are not culturable, and so the use of traditional microbiology techniques cannot provide a full genetic profile of the microbiome. Metagenomic sequencing using next-generation sequencing (NGS) is a powerful method to quantitatively characterize oral microbiomes [22]. The genetic material is recovered directly from samples and due to high-throughput and advancements in bioinformatics, a culture-independent data analysis can be performed [23]. This can provide deeper insight into microbial populations and discover microbes that are not culturable or may be in relatively low abundance and not detectable using traditional methods [24].

The aim of the present study was to profile the oral microbiome in Queensland free-range koalas and associate its composition with age and periodontal disease status (healthy, gingivitis and periodontitis). This is the first study conducted on the oral microbiome of Queensland free-range koalas. Metagenomic studies such as this will advance knowledge on the connection between microbiota and disease in the koala. Future research based on this groundwork will help towards enabling the species to survive and not become extinct.

## 2. Materials and Methods

### 2.1. Ethical Approval

Ethical approval for this study was obtained from the University of Queensland Ethics Committee (Approval Number: DENT/618/UQJRS).

### 2.2. Animal Selection and Age Determination

The koalas were free-range animals in the care of Moggill Koala Hospital, Brisbane, Queensland. Eight koalas were examined for the current oral health status. Seven adults were examined while under general anesthetic. A joey was quickly examined while awake. The koalas were being assessed and medically treated by a veterinarian employed by the Moggill Koala Rehabilitation Centre. All monitoring of the koalas’ health under anesthetic was performed by the veterinarian. The koalas were initially aged using the method of Jackson [25] where koalas are grouped according to the abrasive wear of the right maxillary premolar and molar cusps originally created by Gordon [26]. This determines a tooth wear class (TWC) that is an average estimation of each koala’s age. For microbial analysis the koalas were allocated into 4 groups: joey (up to 9 months), juvenile (TWC 2- 3), adult (TWC 5-6) and old (TWC 7-9).

### 2.3. Clinical Examination and Sample Collection

The koalas were examined for the presence of overall oral health, periodontal disease and any clinical evidence of chlamydial disease symptoms. PD was assessed according to the methodology developed by Pettett, McKinnon, Wilson, Carrick, Sly and Bird [5]. PD was separated into either the presence of gingivitis or periodontitis at four sites (mesial, buccal, distal, lingual). Gingivitis was identified by the presence of bleeding on probing and inflammation of the gingiva around the tooth with each category graded 0 (nil), 1 (mild), 2 (moderate) or 3 (severe). Periodontitis was declared when there was evidence of gingivitis and gingival attachment loss which may have included the presence of a gingival pocket or bone loss around the tooth attachment site. Gingival attachment loss was recorded when gingival probing depth was greater than 2 mm according to AVDC [27], Caiafa [28] and Pettett, McKinnon, Wilson, Carrick, Sly and Bird [5].

Plaque samples were taken from the gingival margins in the front upper and lower incisors, using a curette. Pooled samples were placed in cryovials containing trypticase soy broth containing 10% glycerol. Vials were placed immediately in dry ice, and the samples were frozen for transport to the laboratory and stored in liquid nitrogen. Samples were thawed for DNA extraction.

### 2.4. DNA Extraction and Verification

Bacterial DNA was extracted from each plaque sample using the Powesoil DNA Isolation Kit (Mobio Laboratory, Carlsbad, CA, USA), following the manufacturer’s instructions. The extracted DNA was verified and quantified using a Qubit Fluorometer with the dsDNA BR Assay kit (Thermo Fisher Scientific, Waltham, MA, USA).

### 2.5. 16S rRNA Gene Amplification, Library Preparation and Sequencing

The 16S rRNA gene encompassing the V6 to V8 regions was targeted using the 803F (5′-TTAGAKACCCBNGTAGTC-3′) and 1392R (5′-ACGGGCGGTGWGTRC-3′) primers [29] modified at the end to contain Illumina specific adapter sequence (803F:5′TCGTCGGCAGCGTCAGATGTGTATAAGAGACAGTTAGAKACCCBNGTAGTC3′ and 1392wR: 5′GTCTCGTGGGCTCGGGTCTCGTGGGCTCGGAGATGTGTATAAGAGACAGACGGGCGGTGWGTRC3′).

16S library preparation workflow from Illumina (#15044223 Rev.B, Australian Centre for Ecogenomics, The University of Queensland, Brisbane, Australia) was employed. Initially, PCR products of ~590 bp were amplified according to the specified workflow with an alteration in polymerase used to substitute Q5 Hot Start High-Fidelity 2X Master Mix (New England Biolabs, Notting Hill, VIC, Australia) in standard PCR conditions. DNA purification of PCR products at each stage was performed using Agencourt AMPure XP beads (Beckman Coulter, Lane Cove, NSW, Australia). Purified DNA was indexed with unique 8 bp barcodes using the Illumina Nextera XT v2 Index Kit A-D (Illumina FC-131-1002) in standard PCR conditions with Q5 Hot Start High-Fidelity 2X Master Mix. Indexed amplicons were pooled together in equimolar concentrations and sequenced on MiSeq Sequencing System (Illumina) using paired-end sequencing with V3 300 bp chemistry in the Australian Centre for Ecogenomics (The University of Queensland, Australia) according to the manufacturer’s protocol.

### 2.6. Taxonomic Assignment and OTU Clustering

Adapter trimming, fixed length trimming, merging paired reads and filtering based on the number of reads (to remove the samples with low coverage) were performed to obtain high-quality sequence reads with enough depth for microbiota profiling and comparison. CLC Microbial Genomics Module Version 11 (QIAGEN Aarhus A/S, Aarhus, Denmark) was used to assign taxonomy to the reads from different samples, as previously described. To this end, reads were clustered using representative sequences of pseudo-species called OTUs (operational taxonomic units). The OTU clustering tool clusters the reads and reduces the read collection in each sample to representative sequences (cluster centroids) that are 94% similar to any member of the cluster they represent. The Greengenes2 [30] database was used as the reference database of 16S rRNAs and mapping. The number of reads assigned to each OTU and the relative abundance of each OTU were calculated. The median of relative abundance was calculated at both the phylum and genus level to provide a robust representation of the microbiota composition, given the limited sample size and the typical Poisson distribution of microbiome data.

### 2.7. Statistical Analysis

The effect of age on the oral microbiome was evaluated by the comparison of oral microbiota between joey, juveniles, adults and old koalas at the phylum and genus level. Joey and juvenile were classified as younger koalas while adult and old koalas were classified as older koalas. A Permutational Multivariate Analysis of Variance (PERMANOVA) test was employed to find the significance of change in microbiome composition. Alpha diversity was evaluated based on the Shannon Index using the Kruskal–Wallis H test and Mann–Whitney U test. Beta diversity was examined using Principal Coordinate Analysis (PCoA) and the PERMANOVA test, as described previously [19].

For differential abundance analysis of bacterial taxa, a generalized linear model (GLM) with a Negative Binomial distribution was applied. Significance testing was performed using the Wald test, followed by Benjamini–Hochberg false discovery rate (FDR) correction. Taxa with an adjusted FDR *p*-value < 0.05 were classified as differentially abundant between groups. This analysis was implemented in DESeq2 (Version 1.47.5), which robustly normalizes sequencing depth variations and accounts for variability in count data, making it ideal for analyzing microbiome data [31]. To validate the findings from the Wald test, a complementary proportion-based test using Fisher’s Exact Test was performed. This test was applied to the raw count matrix of reads mapped to taxa. This non-parametric approach provides complementary evidence for differential abundance, particularly for low-count taxa where parametric assumptions may be violated. DESeq2 analysis was conducted in the R environment, while Fisher’s Exact Test was performed using the scipy.stats module within a Python environment in Jupyter Notebook (Version 7.0.8).

To investigate the relationship between koala age and PD with the relative abundance of selected oral bacterial genera, a correlation analysis was performed. Koalas were categorized into four age groups (Joye, Juvenile, Adults and Old koalas) and encoded as ordinal values from 1 (youngest) to 4 (oldest). Eight koalas were classified as either healthy (K1–K4) or having PD (K5–K8), and this status was encoded as a binary variable (0 = healthy, 1 = PD). Six genera of interest (*Fusobacterium*, *Porphyromonas*, *Moraxella*, *Prevotella*, *Acinetobacter* and *Streptococcus*) were selected. Spearman’s rank correlation coefficient was calculated to assess associations between age and the relative abundance of each genus. The resulting *p*-values were recorded where *p*-value < 0.05 was considered statistically significant. *p*-values were recorded, and associations with *p* < 0.05 were considered statistically significant. All analyses were performed in Python (v3) using the pandas and scipy libraries within the Jupyter Notebook environment.

## 3. Results

### 3.1. General and Oral Health

The study consisted of five males and three females with TWCs ranging from joey (≤9 months) to old. Koala No. 1, a joey, was the backrider of Koala No. 5. Five koalas visibly had chlamydial symptoms involving keratoconjunctivitis (Table 1).

**Figure 1:**
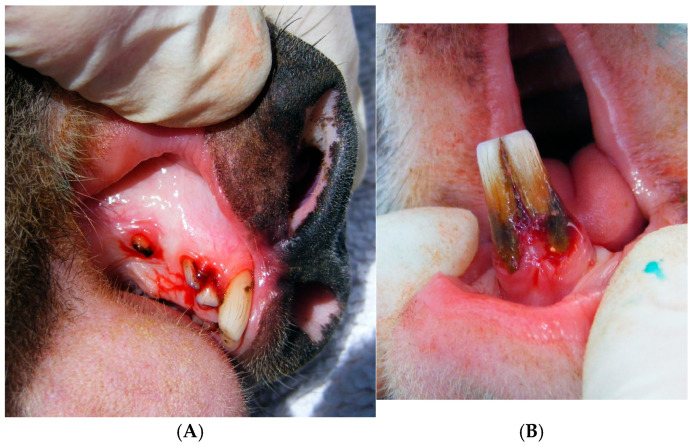
Clinical periodontal disease in koalas. (**A**) Severe (grade 3) bleeding of the maxilla of Koala 8 (TWC 9), accompanied by periodontal pockets and gingival recession. (**B**) Severe (grade 3) inflammation and bleeding (gingivitis) of the mandibular incisors of Koala 8 (TWC 9), also showing gingival recession, periodontal pocket and attachment loss.

Oral health declined with age as shown by increases in scoring levels and severity levels. The full oral health assessment of all koalas can be found in Tables S1–S3 (Supplementary Material). From the oral health assessments, gingivitis (inflammation and bleeding) was present in five koalas and periodontitis (periodontium category in the oral health chart) in four koalas. Four adult koalas aged between TWC 5 and 9 had gingival recession and attachment loss ranging between 7 and 19 mm with compacted vegetation at the attachment loss sites. The lower incisors recorded the highest number of koalas with pockets and the highest frequency of gingival recession (*n* = 6). The upper incisors followed this pattern with five koalas with pockets and two with recession. The canines and upper first, second and third molars all recorded single cases of periodontal pockets in the oldest koala (TWC 9), this koala also recorded pockets in the upper and lower incisors (Figure 1). The male TWC 3 koala was the youngest to record a mild pocket measuring 4 mm in depth.

### 3.2. Phylum-Level Oral Microbiome Composition

**Figure 2:**
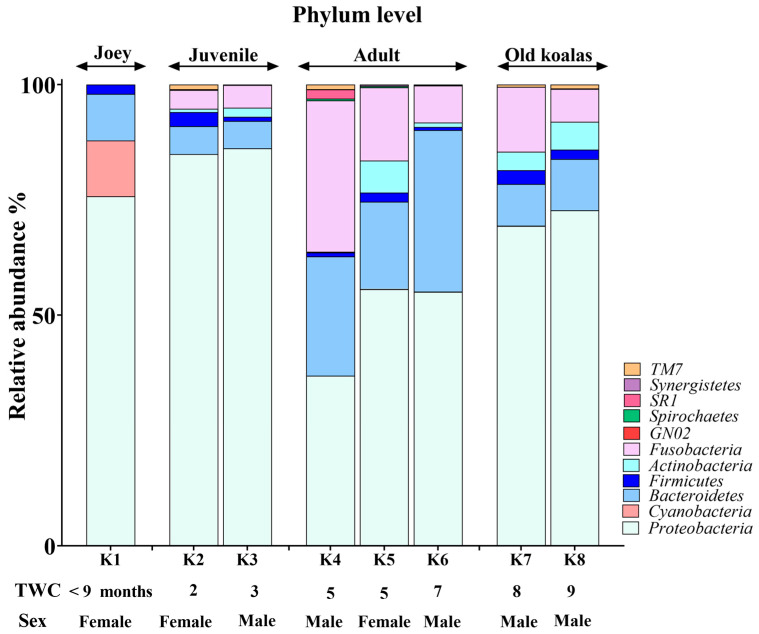
Phylum-level composition of the koala oral microbiome.

**Figure 3:**
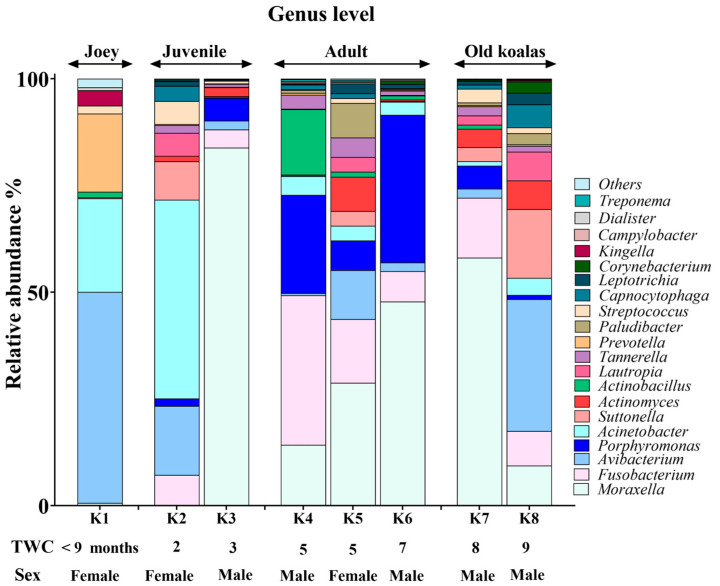
Genus-level composition of the koala oral microbiome.

The koala oral cavity included microbiota from 11 phyla (Figure 2) and 29 genera (Figure 3). The most prominent microbiota came from four phyla, including Proteobacteria, Bacteroidetes, Fusobacteria and Firmicutes, with a relative abundance median of 70.5%, 10.5%, 7.5% and 2%, respectively.

### 3.3. Genus-Level Oral Microbiome Composition

**Figure 4:**
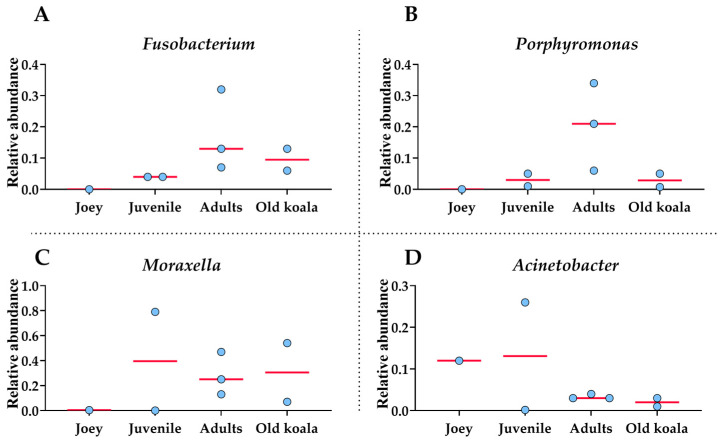
Shifts in oral bacterial genera with age in koalas. (**A**): *Fusobacterium*. (**B**): *Porphyromonas*. (**C**): *Moraxella*. (**D**): *Acinetobacter*. Each circle represents an individual koala. The red lines indicate the medians.

*Moraxella*, *Fusobacterium*, *Avibacterium*, *Porphyromonas* and *Acinetobacter* with median relative abundances of 19%, 6.5%, 5.5% and 5% were the most abundant genera identified (Figure 3). Interestingly, *Fusobacterium* and *Porphyromonas* genera were not detected in the joey oral microbiota (Figure 4). The beneficial genus *Lactobacillus* was only detected in the oral microbiota of the joey, accounting for 0.36% of the total microbial community. It was absent from the oral microbiota of juvenile, adult and old koalas.

### 3.4. Age-Related Shifts in the Oral Microbiome

A number of shifts in bacterial communities occurred with age. In the younger koalas (joey and juvenile), Proteobacteria was well represented (Figure 2). The joey oral cavity contained only four phyla, Proteobacteria (75%), Cyanobacteria (12%), Bacteroidetes (10%) and Firmicutes (2%). Both juveniles’ oral cavities had a wider profile of organisms (Figure 2 and Figure 3), compared to the joey. The adults and old koalas had similar frequencies of microbiota and similar profiles. The adult oral cavities had the highest diversity of phyla.

At the phylum level, the abundance of Proteobacteria decreased with age. In contrast, the relative abundances of Bacteroidetes and Fusobacteria increased in the oral microbiota as koalas aged (Supplemental Data). These differences were statistically significant between younger (joey and juvenile) and older (adult and old) koalas, as determined by the Wald test: Fusobacteria (FDR-adjusted *p*-value < 0.0001), Proteobacteria (FDR-adjusted *p*-value < 0.01) and Bacteroidetes (FDR-adjusted *p*-value < 0.01).

At the genus level, *Fusobacterium* and *Porphyromonas* had remarkably higher abundances in older (adult and old) koalas compared to younger ones (joey and juvenile) (Figure 4), as shown in Figure 4. The differences were highly significant for both *Fusobacterium* and *Porphyromonas* (FDR-adjusted *p*-value < 0.00001, Fisher Exact test). *Acinetobacter* abundance reduced in proportion by the adult stage of life (Figure 4). *Moraxella* abundance was low in the joey (Figure 4).

*Fusobacterium* exhibited a statistically significant positive correlation with age (72%, *p* = 0.045), indicating that its relative abundance increases with aging in koalas. This finding suggests a potential age-associated enrichment of *Fusobacterium* within the oral microbiome. *Porphyromonas* also showed a positive correlation with age (35%, *p* = 0.40), although this association was not statistically significant.

### 3.5. Oral Microbiome Composition in Koalas with Gingivitis and Periodontitis

**Figure 5:**
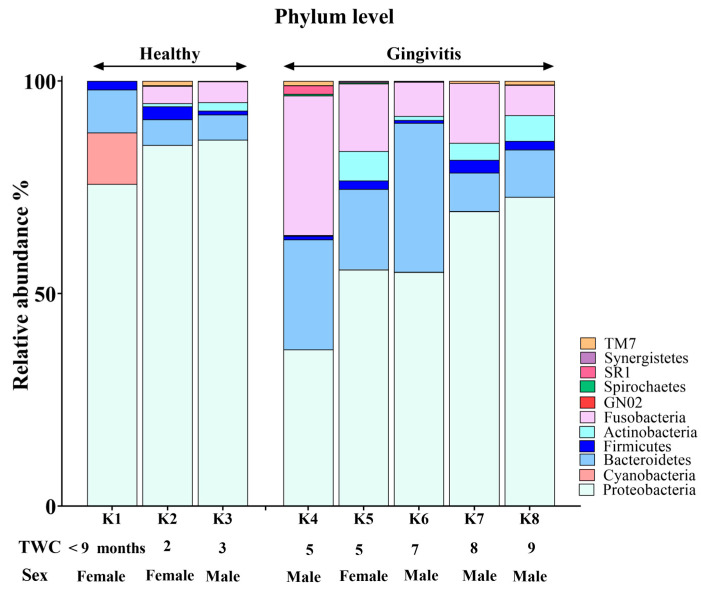
Comparison of oral microbiota at the phylum level between koalas with gingivitis *(n* = 5) and those without gingivitis (*n* = 3).

**Figure 6:**
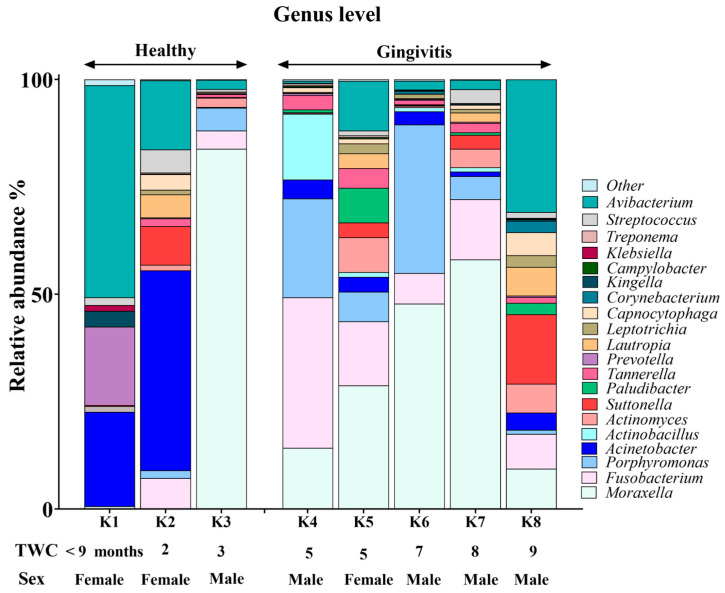
Genus-based comparison of oral microbiota between koalas with gingivitis (*n* = 5) and those without gingivitis (*n* = 3).

Younger koalas (joey and juvenile, *n* = 3) did not show gingivitis. In contrast, older koalas (adult and old ones, *n* = 5) had gingivitis (Figure 5 and Figure 6). Consequently, at the phylum level, a significant decrease in Proteobacteria abundance (FDR-adjusted *p*-value < 0.01), coupled with a significant increase in Bacteroidetes (FDR-adjusted *p*-value < 0.01) and Fusobacteria (FDR-adjusted *p*-value < 0.0001), was the major change in the oral microbiota of koalas with gingivitis (Figure 5). At the genus level, significant increases in the abundances of *Fusobacterium* (FDR-adjusted *p*-value < 0.00001) and *Porphyromonas* (FDR-adjusted *p*-value < 0.00001) were the major shifts in the oral microbiota of koalas with gingivitis.

Koalas with periodontitis (*n* = 4) exhibited an oral microbiota profile that closely resembled that of koalas with gingivitis characterized by a markedly increased abundance of both *Fusobacterium* and *Porphyromonas*.

Correlation analysis showed a moderate negative correlation between the relative abundance of *Acinetobacter* with PD (−44%), suggesting that its relative abundance tends to decrease in koalas with PD. In contrast, *Fusobacterium* and *Porphyromonas* demonstrated moderate positive correlations with PD (44% and 33%, respectively), indicating a tendency for an increased abundance of these genera in koalas affected by PD. Although none of these associations reached statistical significance, the observed trends are biologically suggestive and consistent with the known roles of these taxa in oral health and disease. Further studies with larger sample sizes are needed to validate these findings and clarify the role of these genera in koala periodontal health.

### 3.6. Bacterial Diversity in Oral Koala Microbiome

**Figure 7:**
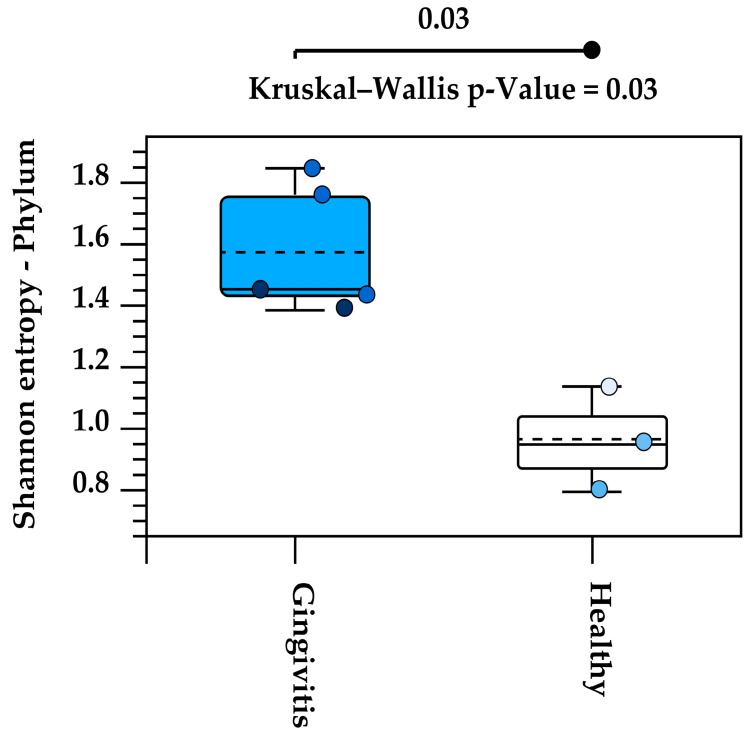
Increased alpha diversity at the phylum level in koalas with gingivitis compared to those without gingivitis. Each circle represents the Shannon entropy of an individual koala at the phylum level. The dashed line inside each box represents the mean value of Shannon entropy for each group (Gingivitis and Healthy). The solid line within the box indicates the median of Shannon entropy.

Alpha diversity analysis of the eight studied oral microbiota samples revealed a high level of within-individual variation associated with age (Supplemental Data). Koalas with gingivitis (*n* = 4) exhibited significantly higher alpha diversity at the phylum level, as determined by the Kruskal–Wallis H test (*p* = 0.03) (Figure 7).

**Figure 8:**
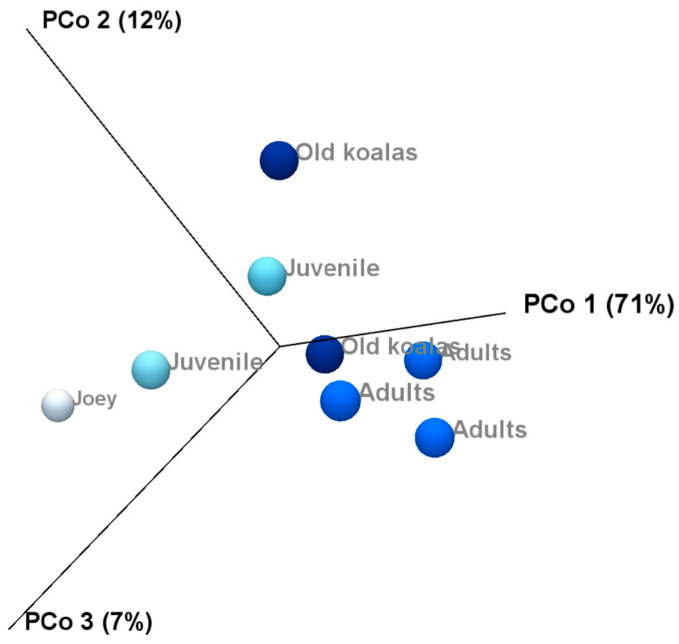
Principal Coordinate Analysis revealed distinct clustering of younger (joey and juvenile) and older (adult and old) koalas based on oral microbiota composition.

Beta diversity and compared microbiota compositions across different age groups shown on the PCoA plots (Figure 8) revealed a clear separation between joey and juvenile koalas (younger group) and adult and old koalas (older group), suggesting distinct microbiota compositions between these age categories. However, PERMANOVA analysis based on the Bray–Curtis distance metric did not yield statistically significant differences between the groups.

## 4. Discussion

Periodontal disease is frequently observed in free-range koalas. The frequency and severity of periodontal disease increases as a koala ages [6]. Within the oral cavity, the molars and incisors are common areas of periodontium destruction [3,32]. Microorganisms from the koala oral cavity have been identified as causing infections in humans [33]. There is growing evidence that there is a link between oral bacteria composition and systemic disease [34,35,36]. In this study, the periodontal plaque of the incisors of eight free-range koalas of various ages was analyzed, comparing the microbiota composition between animals with good oral health and the ones with periodontal disease.

Based on the findings from this study, the oral microbiome of Queensland’s free-ranging koalas exhibits clear compositional changes associated with both age and periodontal disease status. At the phylum level, Proteobacteria emerged as the most dominant phylum in younger individuals, particularly joeys, but its abundance declined with age. In contrast, older koalas showed an increase in Bacteroidetes, Fusobacteria and Actinobacteria, suggesting a progressive microbial shift as koalas mature. The genus-level analysis further emphasizes this disease-associated dysbiosis. *Fusobacterium* and *Porphyromonas* were significantly more abundant in older koalas and those with clinical signs of gingivitis and/or periodontitis. *Fusobacteria* and *Porphyromonas* are commonly associated with oral inflammation and diseases [19,37,38,39,40]. These findings support the hypothesis that aging and periodontal conditions are accompanied by a shift toward pro-inflammatory bacterial taxa [41]. A study involving captive koalas indicated similarity between two koalas’ oral cavity microbiomes [18] which differs from this study where there were shifts in individual microbiota between the age groups and oral disease status.

The genus of *Lactobacillus* was only detected in the oral microbiota of the joey. This genus is found in milk and is evidence that lactation was still occurring [42,43]. *Lactobacillus* is also often linked to good oral health and microbial homeostasis [44]. The phylum Cyanobacteria presence on eucalyptus leaves indicates that the backriding joey was transitioning over to a eucalyptus diet [45]. This aligns with the known decrease in milk ingestion and increase in eucalyptus ingestion [43]. Its complete absence of both *Lactobacillus* and Cyanobacteria in juvenile, adult and old koalas may reflect a loss of protective microbial communities as koala ages, possibly increasing susceptibility to colonization by pathogenic genera.

Increasing age was found to be the main risk factor for the prevalence and severity of PD in both South Australian koalas [6] and Queensland koalas [3]. In our previous study on South Australian koalas, regression analysis revealed a 17% linear increase in gingivitis and 19% linear increase in periodontitis observed by increasing age (R^2^ for gingivitis = 90.64% and R^2^ for periodontitis = 91.84%). The present study further revealed that aging in koalas is associated with significant shifts in oral microbiota composition. Notably, Joey displayed a distinct microbial profile, lacking pathogenic genera such as *Fusobacterium* and *Porphyromonas* and exhibiting the presence of beneficial *Lactobacillus*. In contrast, older (adult and old) koalas had significantly (FDR-adjusted *p*-value < 0.00001) higher abundances of *Fusobacterium* and *Porphyromonas* in their microbiota compared to younger ones (joey and juvenile). Correlation analysis indicated a strong positive relationship between age and the abundance of *Fusobacterium* (72%) and a moderate correlation for Porphyromonas (35%). *Fusobacterium* and *Porphyromonas’* upward trend in abundance with age may reflect a biologically relevant risk pattern in oral health.

Alpha diversity analysis revealed substantial variation in microbiota composition between individual koalas, with diversity patterns influenced by age. Notably, samples from koalas with gingivitis exhibited higher phylum-level diversity compared to healthy individuals. This may indicate microbial overgrowth or shifts in community structure that accompany inflammatory conditions. Beta diversity analysis also revealed distinct clustering of microbial communities by age, with joey and juvenile samples forming a separate cluster from adult and old individuals. However, the lack of statistical significance, likely due to limited sample sizes, suggests that future research with more koalas is needed to confirm the observed trends and associations in this study. Increasing the number of individuals per group would enhance the statistical power of the PERMANOVA test and may uncover more robust group differences. Variation in the individual oral microbiota may show similar trends indicated in koala genetics and gut microbiota variation when based on environmental location [46,47].

Another limitation of our study was the use of partial 16S rRNA gene sequencing, which mainly restricts taxonomic resolution to the genus level. This is particularly important in microbiome studies, where species-level resolution can have significant biological and clinical implications. Full-length 16S rRNA sequencing using long-read technologies such as PacBio HiFi has been shown to provide significantly greater depth and taxonomic resolution, enabling accurate species-level microbiota profiling and uncovering a broader diversity of bacterial taxa [48]. Therefore, future studies of the oral koala microbiome should consider incorporating full-length 16S rRNA sequencing approaches to enhance taxonomic resolution and more accurately capture the complexity of the microbial community.

Studying free-range koalas through a rehabilitation facility would allow further study into the oral microbiome and any relationship between particular browse [46], a limitation of captive studies [49]. This could be vital in understanding colonies that collapse due to local overabundance, drought or loss of vegetation [50]. Koalas with severe PD would have a reduced lifespan, as the consumption of eucalyptus leaves requires optimal dentition health [51]

Genetic and inherited susceptibility towards periodontitis has been identified in mammals [52], koalas having a genome that contains the KorV virus [53] may already have a predisposing factor towards periodontium inflammation, the primary immune response causing periodontitis. Koalas with a chlamydial infection already have a proinflammatory response occurring [1]. This may reduce their ability to stop gingivitis from increasing in severity resulting in periodontitis. Additionally, the oral cavity is regarded as the gateway to detecting oral and systemic diseases, and any population screening of the oral cavity that indicates dysbiosis is occurring may aid in the discovery of any trends towards poor population health [54]. Periodontium destruction could place the koala at risk of hematological transmission of infections. Any links between PD and infections such as chlamydiosis and the KoRV would identify oral-systemic connections.

## 5. Conclusions

Overall, this study represents the first detailed characterization of the oral microbiome of Queensland’s free-ranging koalas and reveals a strong association between microbiome composition, host age and periodontal health. The enrichment of pathogenic genera in older and diseased koalas, particularly *Fusobacterium* and *Porphyromonas*, alongside the disappearance of beneficial microbes like *Lactobacillus*, underscores the potential for using microbiome profiling as a diagnostic or monitoring tool in koala health management. These findings contribute to a growing body of literature on the importance of oral microbiota in wildlife health and may have implications for conservation efforts and veterinary care protocols.

## Figures and Tables

**Table 1:** Clinical grades of chlamydiosis conditions.

Koala	Sex	TWC	Conjunctivitis Severity		Cystitis Severity	Outcome
			Left Eye	Right Eye		
1	F	9 months	0	0	0	no treatment/released
2	F	2	3	3	0	treated/released
3	M	3	1, injured eye	0	0	treated/released
4	M	5	3	3	0	treated/released
5	F	5	3	3	0	treated/released
6	M	7	3	3	0	treated/released
7	M	8	blind/damaged eye	0	0	treated/released
8	M	9	1	1	0	treated/released

Chlamydiosis conditions were graded by observations of the ocular and urogenital sites using scoring of 0 (nil), 1 (mild), 2 (moderate) and 3 (severe) according to Queensland Dept. Env. & Science Moggill Koala Hospital Records.

## Data Availability

The raw sequencing data generated in this study is openly available in the Zenodo data repository (https://zenodo.org/records/15660572 (accessed on 14 June 2025) in FASTQ format, DOI 10.5281/zenodo.15660571. These data include all demultiplexed sequence files used for taxonomic profiling and differential abundance analysis as described in the Methods section.

## Supplementary Materials

The following supporting information can be downloaded at: https://www.mdpi.com/article/10.3390/ani15131834/s1, Table S1: Presence of visual chlamydial symptoms, gingivitis, and periodontitis. 0 = absent; 1 = present; Table S2: Oral health scores from koala oral health chart; Table S3. General oral cavity scores from koala oral health chart.
