# Supplementary material for: The Oral Microbiome in Queensland Free-Ranging Koalas (Phascolarctos cinereus) and Its Association with Age and Periodontal Disease
Source: Animals (Basel). 2025 Jun 20;15(13):1834. doi: 10.3390/ani15131834 (PMC12249121; doi:10.3390/ani15131834)
Supplement: Supplementary file 1 [file animals-15-01834-s001.zip › animals-3659477-supplementary.pdf]

Supplementary Material

The oral microbiome in Queensland free-ranging koalas (*Phascolarctos cinereus*) and its association with age and periodontal disease

Contents

Table S1. Presence of visual chlamydial symptoms, gingivitis, and periodontitis. 0 = absent; 1 = present 1

Table S2. Oral health scores from koala oral health chart 2

Table S3. General oral cavity scores from koala oral health chart 2

Table S1. Presence of visual chlamydial symptoms, gingivitis, and periodontitis. 0 = absent; 1 = present

Koala	Sex	TWC or Age*	Age Group	Chlamydia	Gingivitis	Periodontitis
1	F	9 months*	Young	0	0	0
2	F	2	Juvenile	1	0	0
3	M	3	Juvenile	0	0	0
4	M	5	Adult	1	1	0
5	F	5	Adult	1	1	1
6	M	7	Adult	1	1	1
7	M	8	Old	0	1	1
8	M	9	Old	1	1	1

Periodontal disease was categorised as either gingivitis or periodontitis. Gingivitis was identified by the presence of bleeding on probing and inflammation of the gingiva around a tooth site. Periodontitis was diagnosed when there was evidence of gingivitis along with gingival attachment loss, which could include the presence of a gingival pocket or bone loss around the tooth attachment site.

Chlamydia was considered present if keratoconjunctivitis or wet bottom was observed. The absence of symptoms does not necessarily indicate that a koala was free of chlamydia, as many cases are visually asymptomatic.

Table S2. Oral health scores from koala oral health chart

**Koala**	**1**	**2**	**3**	**4**	**5**	**6**	**7**	**8**
**Sex**	f	f	m	m	f	m	m	m
**TWC**	9 mths	2	3	5	5	7	8	9
Calculus	0.00	0.00	0.86	1.43	1.71	1.57	2.00	2.43
Bleeding	0.00	0.00	0.00	0.86	1.00	1.29	1.57	1.72
Inflammation	0.00	0.00	0.00	0.57	1.57	1.29	1.57	1.57
Mobility	0.00	0.00	0.00	0.00	0.43	0.14	0.00	0.43
**OHI Index 0-12**	0.00	0.00	0.86	2.86	4.71	4.29	5.14	6.15
**Gingivitis Index 0-3**	0.00	0.00	0.00	0.71	1.29	1.29	1.57	1.64
Shading severity represents	= mild	= moderate	=severe					
Gingivitis Presence	0	0	0	1	1	1	1	1
Periodontitis Presence	0	0	0	0	1	1	1	1

Table S3. General oral cavity scores from koala oral health chart

**Koala**	**1**	**2**	**3**	**4**	**5**	**6**	**7**	**8**	**Total**
**Sex**	f	f	m	m	f	m	m	m	
**TWC**	9 mths	2	3	5	5	7	8	9	
Periodontium	0	0	0	0	1	1	1	1	4
Breath	0	0	0	0	1	1	0	0	2
Pockets	0	0	1	1	1	1	1	1	6
Molar Wear	0	0	0	1	1	0	0	0	2
Incisor Wear	0	0	0	1	0	0	0	0	1
Occlusion	0	0	0	1	0	0	1	0	2
Vegetation Compaction	0	0	0	0	1	1	1	1	4
Hard Palate Irregularity	0	0	0	0	0	1	1	0	2
Cheek Irregularity	0	0	0	0	0	0	0	0	0
Tooth Irregularity	0	1	0	0	1	1	0	1	4
Stain Deposit	0	0	0	0	0	0	1	1	2
Tongue Irregularity	0	0	0	0	0	0	0	0	0
**GOCI Total**	0	1	1	4	6	6	6	5	
**GOCI+OHI index**	0.00	1.00	1.86	6.86	10.71	10.29	11.14	11.14	
Scores are 0=not present, 1=condition is present, GOCI score is out of 12.									
Shading severity represents	= mild	= moderate	= severe						
